# Prevention of venous thromboembolic events in patients with lower leg immobilization after trauma: Systematic review and network meta-analysis with meta-epsidemiological approach

**DOI:** 10.1371/journal.pmed.1004059

**Published:** 2022-07-18

**Authors:** D. Douillet, C. Chapelle, E. Ollier, P. Mismetti, P.-M. Roy, S. Laporte

**Affiliations:** 1 Emergency Department, Angers University Hospital, Health Faculty, Angers, France; 2 UNIV Angers, UMR MitoVasc CNRS 6015 –INSERM 1083, Equipe CARME, Angers, France; 3 F-CRIN INNOVTE network, Saint-Etienne, France; 4 Clinical Pharmacology Department, University Hospital of Saint-Etienne, Saint-Etienne, France; 5 SAINBIOSE INSERM U1059, Vascular Dysfunction and Hemostasis, Jean Monnet University, Saint-Etienne, France; University of Toronto, CANADA

## Abstract

**Background:**

Lower limb trauma requiring immobilization is a significant contributor to overall venous thromboembolism (VTE) burden. The clinical effectiveness of thromboprophylaxis for this indication and the optimal agent strategy are still a matter of debate. Our main objective was to assess the efficacy of pharmacological thromboprophylaxis to prevent VTE in patients with isolated temporary lower limb immobilization after trauma. We aimed to estimate and compare the clinical efficacy and the safety of the different thromboprophylactic treatments to determine the best strategy.

**Methods and findings:**

We conducted a systematic review and a Bayesian network meta-analysis (NMA) including all available randomized trials comparing a pharmacological thromboprophylactic treatment to placebo or to no treatment in patients with leg immobilization after trauma. We searched Medline, Embase, and Web of Science until July 2021. Only RCT or observational studies with analysis of confounding factors including adult patients requiring temporary immobilization for an isolated lower limb injury treated conservatively or surgically and assessing pharmacological thromboprophylactic agents or placebo or no treatment were eligible for inclusion. The primary endpoint was the incidence of major VTE (proximal deep vein thrombosis, symptomatic VTE, and pulmonary embolism-related death). We extracted data according to Preferred Reporting Items for Systematic Reviews and Meta-analyses for NMA and appraised selected trials with the Cochrane review handbook. Fourteen studies were included (8,198 patients). Compared to the control group, rivaroxaban, fondaparinux, and low molecular weight heparins were associated with a significant risk reduction of major VTE with an odds ratio of 0.02 (95% credible interval (CrI) 0.00 to 0.19), 0.22 (95% CrI 0.06 to 0.65), and 0.32 (95% CrI 0.15 to 0.56), respectively. No increase of the major bleeding risk was observed with either treatment. Rivaroxaban has the highest likelihood of being ranked top in terms of efficacy and net clinical benefit. The main limitation is that the network had as many indirect comparisons as direct comparisons.

**Conclusions:**

This NMA confirms the favorable benefit/risk ratio of thromboprophylaxis for patients with leg immobilization after trauma with the highest level of evidence for rivaroxaban.

**Trial registration:**

PROSPERO CRD42021257669.

## Abstract 2

Delphine Douillet and colleagues investigate the benefit/risk ratio of thromboprophylaxis for patients with leg immobilization after trauma.

## Author summary

**Why was this study done?:**

- Lower limb trauma requiring immobilization is a significant contributor to overall venous thromboembolism (VTE) burden.
- Existing evidence suggests that pharmacological prophylaxis could reduce overall VTE event rates in these patients, but the proportional reduction of symptomatic events remains unclear as does the treatment that provides the best clinical benefit.
- International guidelines continue to offer conflicting advice for clinicians and thromboprophylaxis strategies vary widely between physicians in different countries.

**What did the researchers do and find?:**

- This study is a systematic review and a Bayesian network meta-analysis including all available randomized trials comparing a pharmacological thromboprophylactic treatment to placebo or no treatment in patients with leg immobilization after trauma.
- We assess the efficacy and safety of 5 different existing strategies (LMWH, fondaparinux, aspirin, rivaroxaban, and control) to compare them against one another and to define the one that appears to be the most efficient with the highest level of evidence.
- Including 14 RCTs and 8,198 patients, rivaroxaban, fondaparinux, and low molecular weight heparins were associated with a significant risk reduction of major VTE.

**What do these findings mean?:**

- This study confirms the efficacy of anticoagulant treatment to prevent VTE events in lower extremity trauma patients. It shows the potential value of rivaroxaban, this treatment having the best efficacy and safety profile.
- Additionally, it recommends that the efficacy of aspirin is uncertain and requires further well-performed investigation.

## Introduction

### Background

Patients with an isolated lower limb trauma requiring immobilization are at increased risk of venous thromboembolism (VTE), which includes deep vein thrombosis (DVT) (proximal or distal) and pulmonary embolism (PE). VTE is a serious and potentially life-threatening complication. In this population including various situations, asymptomatic VTE occurred in 18.0% (95% CI 12.9 to 23.1) and symptomatic VTE in 2.0% (95% CI 1.3 to 2.7) [1,2]. The efficacy and safety of thromboprophylaxis in reducing thromboembolic events in immobilized lower limb trauma patients has been established in several trials [3–6]. However, the POT-CAST study showed no significant effects of low molecular weight heparins (LMWHs) versus no treatment on the rate of symptomatic VTE [3]. Conversely, recent publications assessing direct oral anticoagulants (DOACs) and aspirin suggest that these molecules are effective [6,7]. Nowadays, guidelines on thromboprophylaxis and, therefore, clinical practices vary widely between countries, ranging from no preventive anticoagulation in the United States (unless the trauma is to be managed surgically) [8] to thromboprophylaxis for all patients for whom plantar support is not possible in France [9]. A number of countries, such as England, Germany, and Australia, propose that the risk of thrombosis should be assessed before initiating thromboprophylaxis [10–12]. It has been observed that there is no consensus among clinicians who do not follow the recommendations in a consensual manner in terms of deciding whether initiating thromboprophylaxis or in terms of choosing an antithrombotic molecule. In addition, the recommendations should be updated as new studies have been published [8].

Horner and colleagues published the only network meta-analysis (NMA) pooling the results of 13 randomized controlled trials (RCTs) on this population [13]. It concludes that LMWH and fondaparinux were effective in preventing any VTE in trauma patients requiring thromboprophylaxis. However, this review has some limitations. First, they assessed a 3-arm network with LMWH, fondaparinux and a control group pooling placebo, no treatment, and aspirin. But considering aspirin as an inactive molecule is questionable, given that aspirin is at the same time a recommended thromboprophylaxis for patients with major surgery mainly in the context of elective surgery (i.e., patients undergoing total hip or total knee arthroplasty) in the US [8,14,15]. Aspirin deserves to be singled out as a potential active treatment. Moreover, LMWH and fondaparinux were only compared with a control and not with each other. Second, the level of evidence varies greatly from study to study. Although they have shown no difference in treatment effect in a sensitivity analysis removing 3 studies considered at high risk of bias by the authors, the studies deserve to be analyzed separately according to the risk of bias. In addition, since this NMA was performed, new data are available with DOAC, especially with the publication of the PRONOMOS study, an RCT comparing rivaroxaban versus LMWH for patients with nonmajor surgery [6]. DOACs have proven their efficacy in major orthopedic surgery [16–18], and rivaroxaban, apixaban, and dabigatran are currently recommended for thromboprophylaxis after a total hip or knee replacement and should also be considered as a potential active treatment.

This systematic review coupled with an NMA aims to assess the efficacy of pharmacological thromboprophylaxis to prevent VTE in patients with temporary lower limb immobilization after trauma. Our main objective was to estimate the clinical efficacy and safety of each of the pharmacological thromboprophylaxis options and to compare the molecules to determine the best strategy available. We also assessed the potential modification of the treatment effect according to the risk of bias of the studies using a meta-epidemiological approach.

## Materials and methods

### Study protocol

This study is reported as per the Preferred Reporting Items for Systematic Reviews and Meta-Analyses (PRISMA) guideline [19]. The protocol of the review was registered on the PROSPERO international prospective register of systematic reviews (CRD42021257669).

### Search strategy and selection criteria

An exhaustive literature search, both manual and computer assisted, was performed on all data up to and including July 29, 2021, with no restriction on the non-English languages in which the sources were published. The computer-assisted search was carried out on electronic databases (Medline, Embase), Google Scholar, the Cochrane Library, and the international database of clinical trials (www.clinicaltrials.gov). The following keywords were used: trauma, injury, immobilization, casts, Achilles’ tendon rupture, LMWH, fondaparinux, DOAC, aspirin in combination with controlled or randomized, and trial, study, observational, and cohort study (S1 Table). Reference lists of journal articles, as well as proceedings from major international meetings (International Society on Thrombosis and Haemostasis, International Congress on Thrombosis), were manually reviewed to locate additional studies. Particular attention was paid to the risk of duplicate reports, and whenever identified, duplicate studies were excluded. When studies were published in both abstract form and as a full article, only the full publication was considered. When more than one article was available for the same study, the relevant information from all publications was extracted.

All references were reviewed for potential inclusion by 1 reviewer (DD), and any citations that clearly did not meet the inclusion criteria were excluded. All abstracts and full text articles were then examined independently by 2 reviewers (DD and CC). Any disagreements in the selection process were resolved through discussion or, if necessary, opinion of a third reviewer (SL). Disagreements were resolved by consensus, and all authors agreed on the final selection of included articles.

To be included in the meta-analysis, studies had to meet all the following criteria: (i) RCT or observational study with analysis of confounding factors; (ii) including adult patients requiring temporary immobilization (plaster cast or removable splint) for an isolated lower limb injury treated conservatively or surgically (excluding major polytrauma); (iii) comparing pharmacological thromboprophylactic agents with each other (i.e., LMWH agent, fondaparinux, aspirin, or DOAC) or with placebo or no treatment; and (iv) assessing the rate of VTE (DVT, PE, death related to PE) and/or major bleeding (as defined within each study). Studies published before 1990 were excluded. For studies including patients requiring temporary immobilization after trauma and/or for another reason as elective surgery, only the subgroup of patients with trauma were included in our meta-analysis. In this case, the authors were contacted to retrieve data from trauma patients.

### Data extraction and quality assessment

Two of the authors (DD and CC) independently extracted data concerning study design, study quality, treatment regimens, population characteristics, and efficacy and safety outcomes. The data extracted from each study were reviewed and, in the case of disagreement, confirmed by arbitration by a third reviewer (SL). When data were unavailable or unclear, we attempted to contact the corresponding authors through e-mail and inspected previous systematic reviews for the trial data of interest. Any issues with data extraction were discussed and resolved by consensus.

Three authors (DD, CC, and SL) independently assessed the risk of bias within all included studies according to the Cochrane review handbook using on the Cochrane RoB 2 tool (Version 2 of the Cochrane risk-of-bias tool for randomized trials) taking into account random sequence generation, concealment of the allocation sequence, blinding of participants and personnel, blinding of outcome assessment, incomplete outcome data, and selective reporting, and ROBINS-I (risk of bias in nonrandomized studies—of interventions) for nonrandomized studies [20,21]. We considered studies as having a low risk of bias if the concealment of the allocation was adequate and the assessment of outcomes was blinded. In the case of older studies, there was a lack of protocol data and, therefore, imprecision in this item of the Rob2 tool, so it was not considered in the risk of bias assessment.

### Outcome definitions

The primary efficacy endpoint was major VTE (DVT and/or PE) defined as the composite of symptomatic VTE, asymptomatic proximal DVT, and death related to PE assessed at the follow-up planned in each study. The secondary efficacy endpoint was all symptomatic VTE (DVT and/or PE) defined as reported within individual trials. In studies without systematic assessment of VTE (e.g., with compression leg vein ultrasonography or phlebography), VTE were classified as symptomatic VTE. The primary safety endpoint was major bleeding defined as reported in each study. Due to the inclusion of studies carried out before 2005, it was not possible to apply the criteria defined by the ISTH to define major bleeding [22]. For these studies, when bleeds were reported without specifying severity, they were considered as major bleeding. Finally, we assessed the net clinical benefit of each treatment defined as major VTE or major bleeding.

### Statistical analyses

The control group included no treatment or placebo in the NMA. The different thromboprophylaxis drugs were considered as separate interventions (i.e., LMWH, aspirin, fondaparinux, and DOACs) in the NMA because of their different mechanisms of action. The different types of LMWH agent were pooled and considered as a single intervention. We made 2 network diagrams to illustrate which of the considered treatments (nodes) were compared (connected) directly and which were compared indirectly through one or more common comparators: the first including only RCT considered at low risk of bias and the second that including all RCTs. We conducted a Bayesian NMA with an unconstrained, random-effects model. Bayesian NMAs are commonly used as they naturally produce rankings and allow the use of prior distributions and direct probability statements can be made around Bayesian estimates, whereas frequentist methods rely on repeated sampling and *p*-values to inform conclusions. Events were supposed to follow a binomial distribution. We considered the possibility of a multi-arm study. Treatment effect estimates were presented as odds ratios (ORs) with 95% credible interval (95% CrI). Noninformative prior distributions were used for the model’s parameters. The Gelman–Rubin statistics was used to test convergence [23]. The Gelman–Rubin statistics was checked automatically every set size number of iterations, and once the series had converged, we stored the last half of the sequence. The parameters tested for convergence were the relative treatment effects, baseline effect, and the heterogeneity parameter.

An inconsistency model was used to test the consistency assumption [24]. Inconsistency was evaluated using a node-splitting procedure. The inconsistency estimate and the corresponding *p*-value were reported. If the *p*-value was low, it meant that we could reject the null hypothesis that the direct and indirect evidence were consistent. We repeated this for all pairs in the network to identify pairs that might be inconsistent [25]. The node-splitting procedure was applied for all loops in the network to identify ones that might be inconsistent. We also performed probabilistic analysis and report the results with a surface under the cumulative ranking curve (SUCRA), a numeric presentation of the overall ranking based on the probability that a treatment was most effective for the outcome of interest with the 95% CrI. Only thromboprophylactic agents that have demonstrated their efficacy have been included in this ranking.

We performed a subgroup analysis according to the risk of bias of the studies: low risk of bias, high risk of bias, or some concerns (i.e., with inadequate allocation concealment and/or nonblinded outcome assessment). We assessed the following potential treatment effect modifiers in a series of meta-regressions: age, duration of thromboprophylaxis, type of treatment (conservatively treated or surgical treatment), and the study design. To do so, we added continuous or discrete covariates to fit a network meta-regression. Statistical analyses were performed using R software version 4.0.3 (R Foundation for Statistical Computing) and the following R packages: “bnma,” “rjags,” and “ggplot2” [26,27].

## Results

### Selection of the studies

**Fig 1:**
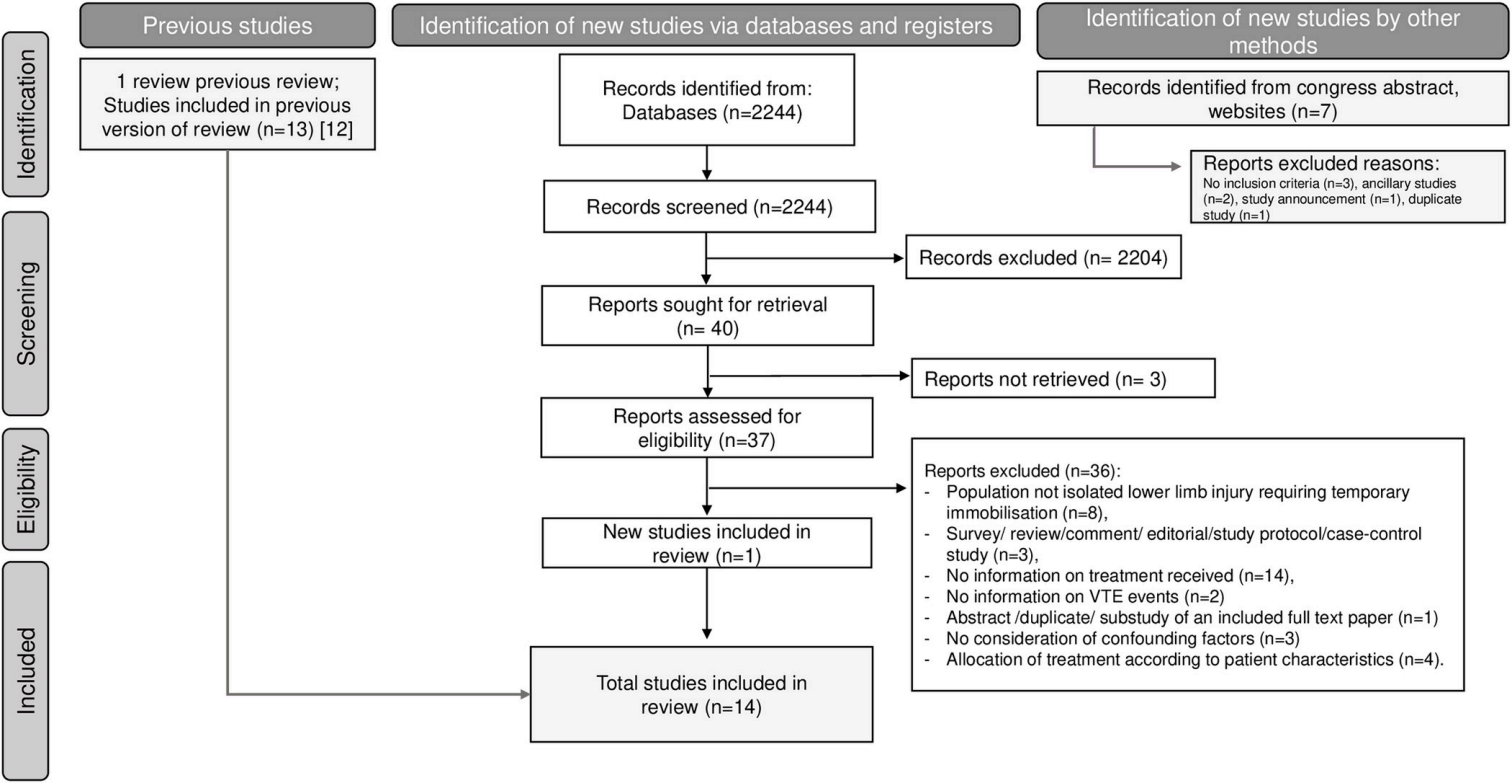
PRISMA flow chart.

The study selection process is presented in Fig 1. The initial search identified 2,251 potentially eligible studies. Fourteen studies met the inclusion criteria. All were RCTs [3–6,28–37] including 8,198 patients. No observational studies were selected because none considered confounding bias in the analysis of the different treatments. Studies excluded after a full text review are listed in S2 Fig.

### Description of included studies

Among the 14 RCTs included (8,198 patients), 7 studies were double blind (3,257 patients) [5,6,31,33–35,37], 4 were open label with a blind evaluation (PROBE [prospective randomized open trial with a blinded evaluation] (3,635 patients)) [3,4,32,36], and 3 were open without blind adjudication (1,306 patients) [28–30] (Table 1). All studies were published between 1993 to 2021. One German-language publication was translated [30]. Table 1 summarizes the key characteristics of the included studies. Thirteen studies assessed a LMWH (mainly dalteparin), 2 fondaparinux, 1 DOAC (rivaroxaban), and 1 aspirin. For 1 study with rivaroxaban involving patients with minor surgery, only the subgroup of patients with trauma were included in the present meta-analysis [6]. For the other studies, we considered the overall included populations. Seven studies focused on fracture or/and Achilles tendon rupture (*n* = 5,530 patients) [4,5,31,33–35,37]. Five studies included patients with conservative treatment (*n* = 2,850) [4,28,29,32,36], 6 with surgical treatments (*n* = 2,817) [5,6,33–35,37], and 3 with patients treated conservatively and/or surgically (*n* = 2,531) [3,30,31]. Finally, 7 studies included all lower limb trauma (i.e., fracture or soft tissue injury) (*n* = 2,668, 3) [6,28–30,32,36].

**Table 1:** Characteristics of included studies and patients’ characteristics.

Author, Year *Study acronym*	Country	Design	Population	Treatment (conservative or surgical)	Intervention	Dose	Duration	Comparators	Assessment of VTE	No. of patients
***Randomized controlled studies***										
Kujath and colleagues, 1993 ^[^^25^^]^	Germany	Open	Patients over 16 years (mean age 34 years; female 42%), Trauma: fracture or soft tissue injury	Conservative treatment	LMWH–Nadroparin	2,850 IU	15 days	No treatment	US after plaster cast removal	306
Kock and colleagues, 1995 ^[^^26^^]^	Germany	Open	Adults (18–75 years; mean age 34 years; female, 39%), Trauma: fracture or soft tissue injury	Conservative treatment	LMWH—certoparin	3,000 IU	14 days	No treatment	US confirmed by venography after plaster cast removal	428
Gehling and colleagues, 1998 ^[^^27^^]^	Germany	Open	Patients over 16 years (mean age 36 years; female 51%), Trauma: fracture or soft tissue injury.	Management approach unclear (mainly surgically treated)	LMWH—Reviparin	1,750 IU	Throughout cast immobilization	Aspirin (1,000 mg/day)	Duplex sonography (all) or phlebography if thrombosis suspected)	572
Lassen and colleagues, 2002 ^[^^28^^]^	Denmark	DB	Adults (>18 years; median age 47 years; female 48%), Trauma: fracture or Achilles tendon rupture.	Conservative or surgical treatment	LMWH—Reviparin	1,750 IU	43 days	Placebo	Unilateral venography after plaster cast removal	440
Jørgensen and colleagues, 2002 ^[^^29^^]^	Denmark	PROBE	Adults (>18 years; mean age 48 years; female 43%), Trauma: fracture or soft-tissue injury.	Conservative treatment	LMWH—Tinzaparin	3,500 IU	38 days	No treatment	Unilateral venography after plaster cast removal	300
Lapidus and colleagues, 2007a ^[^^30^^]^	Sweden	DB	Adults (18–75 years; mean age 40 years; female 21%), Trauma: Achilles tendon rupture.	Surgical treatment	LMWH—Dalteparin	5,000 IU	43 days	Placebo	Unilateral US confirmed by venography 3 and 6 weeks after surgery	105
Lapidus and colleagues, 2007b ^[^^31^^]^	Sweden	DB	Adults (18–75 years; mean age 48 years; female 54%), Trauma: ankle fracture.	Surgical treatment	LMWH—Dalteparin	5,000 IU	44 days	Placebo	Unilateral venography at the end of treatment	272
Goel and colleagues, 2009 ^[^^32^^]^	Canada	DB	Adults (18–75 years; mean age 41 years; female 38%), Trauma: fracture.	Surgical treatment	LMWH—Dalteparin	5,000 IU	14 days	Placebo	Bilateral venography at the end of treatment	305
Samama and colleagues, 2013 ^[^^33^^]^ *FONDACAST study*	France	PROBE	Adults (>18 years; mean age 46 years; female 53.4%), Trauma: fracture or soft tissue injury.0/0/00 0:00:00 AM	Conservative treatment	Fondaparinux	2.5 mg	33.7 days	LMWH–Nadroparin (2,850 IU/day)	Compression ultrasonography and/or venography performed for suspected DVT after cast removal	1,349
Selby et al., 2015 ^[^^34^^]^ *D-KAF study*	Canada	DB	Patients over 16 years (mean age 49 years; female 48%), Trauma: fracture.	Surgical treatment	LMWH—Dalteparin	5,000 IU	14 days	Placebo	Bilateral proximal US at end of treatment	265
Zheng and colleagues, 2016 ^[^^5^^]^	China	DB	Adults (>18 years; mean age 47.8 years; female 37.7%), Trauma: fracture.	Surgical treatment	LMWH–no information	unknown	14 days	Placebo	Blinded bilateral Doppler compression ultrasound	814
Bruntink and colleagues, 2017 ^[^^4^^]^	the Netherlands	PROBE	Adults (>18 years; mean age 47 years; female 58%), Trauma: fracture of the ankle or foot.	Conservative treatment	LMWH—Nadroparin	2,850 IU	40 days	Fondaparinux (2.5 mg/day) or no treatment	Duplex sonography after the removal of the cast	467
Van Adrichem and colleagues, 2017 ^[^^3^^]^ *POT-CAST study*	the Netherlands	Open	Adults (>18 years; mean age 46 years; female 50.1%), Trauma: fracture or soft tissue injury.	Conservative or surgical treatment	LMWH–Nadroparin or Dalteparin	2,850 IU	26 days	No treatment	Symptomatic VTE within 3 months after the procedure. DVT determined by abnormal compression US	1519
Samama and colleagues, 2020 ^[^^6^^]^ *PRONOMOS study*	France	DB	Subgroup analysis: only patients with trauma where selected. Adults (>18 years; mean age 46.1 years; female 36.7%),	Surgical treatment	DOAC—Rivaroxaban	10 mg	37 days	Enoxaparin (4,000 IU/day)	Compression ultrasonography at the end of the immobilization	1,056

DB, double blind; DOAC, direct oral anticoagulant; DVT, deep vein thrombosis; IU, international unit; LMWH, low molecular weight heparin; PROBE, Prospective Randomized Open-label Blinded End-point; US, ultrasound; VTE, venous thromboembolism.

### Quality assessment

The assessments of the risk of bias within each study are summarized in S1 and S2 Figs. Seven RCTs were considered at low risk of bias, i.e., with adequate allocation concealment and blinded outcome assessment (*n* = 3,962 patients) [3,6,29,33–35,37]. For the studies of Jorgensen and colleagues and Lassen and colleagues, information that would have assisted us in assessing bias was, however, missing. However, the authors were contacted during the meta-analysis of Zee and colleagues, enabling them to provide reliable data to assess the risk of bias in their article.

### Network

**Fig 2:**
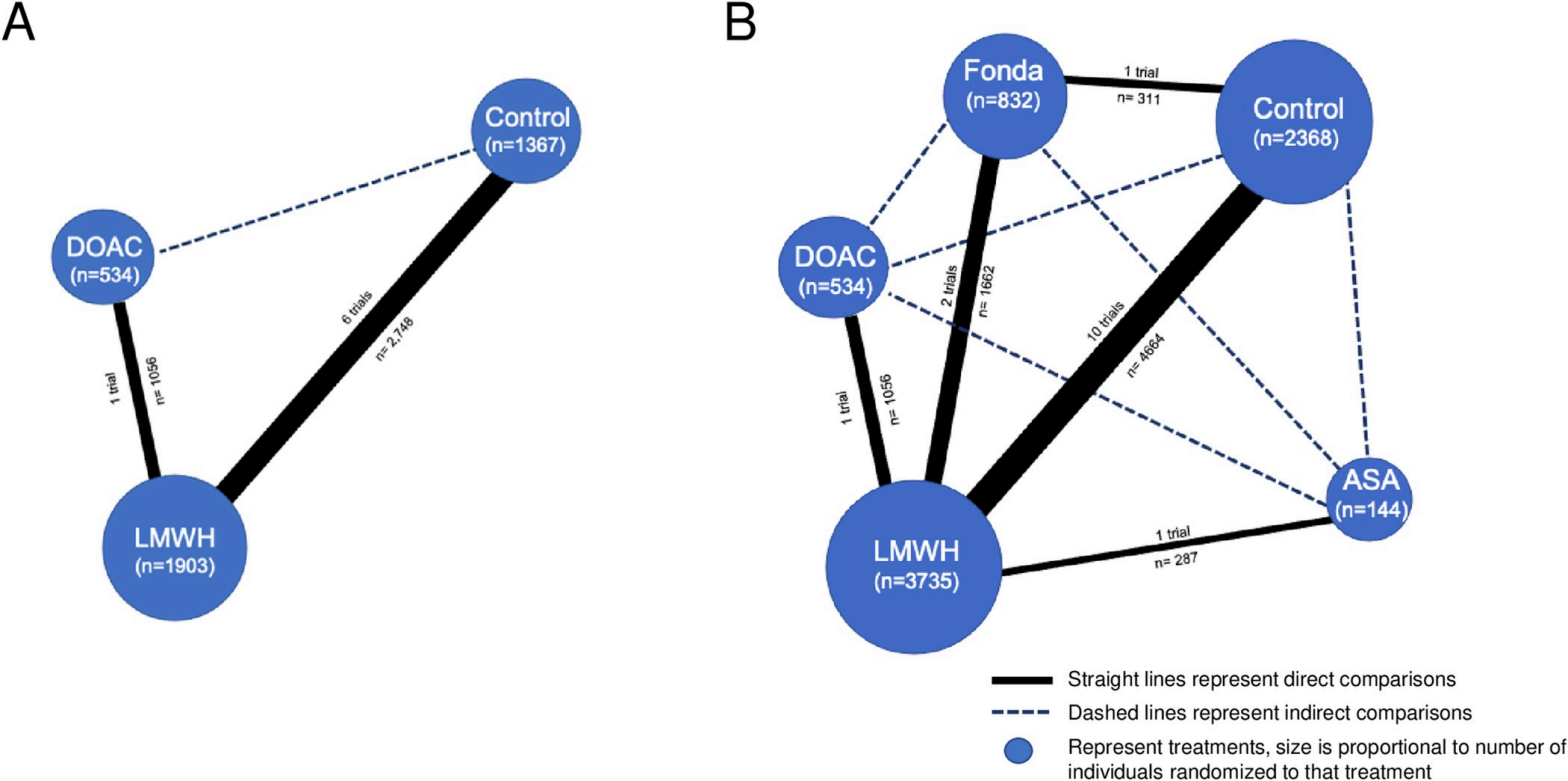
Network graph. (A) Including only RCT with low risk of bias. (B) Including all RCTs. ASA, aspirin; Control, placebo or no treatment; DOAC, direct oral anticoagulant; Fonda, fondaparinux; LMWH, low molecular weight heparin; RCT, randomized controlled trial.

Fig 2A and 2B display the network geometry of low risk of bias RCTs and all RCTs, respectively. The network including all RCTs was composed of a single closed loop consisting of 3 nodes, and 2 other direct comparisons. No studies have assessed other DOACs or warfarin in this indication.

### Efficacy endpoints: Major VTE and symptomatic VTE

**Fig 3:**
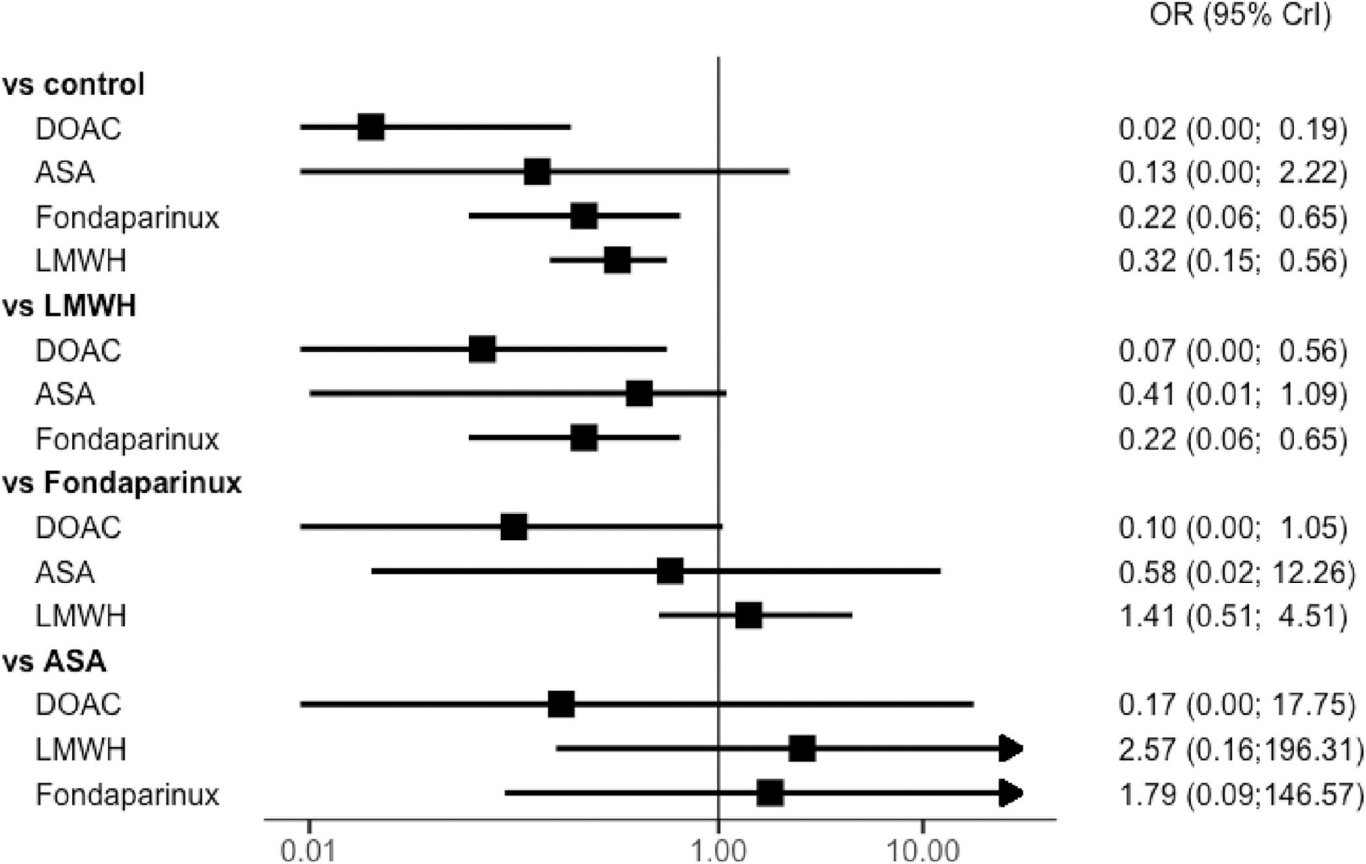
Network forest plot for the primary outcome (major VTE) in all RCTs with OR (points) and their 95% CrIs (lines). ASA, aspirin; DOAC, direct oral anticoagulant; LMWH, low molecular weight heparin; OR, odds ratio; RCT, randomized controlled trial; VTE, venous thromboembolism; 95% CrI, 95% credible interval.

**Fig 4:**
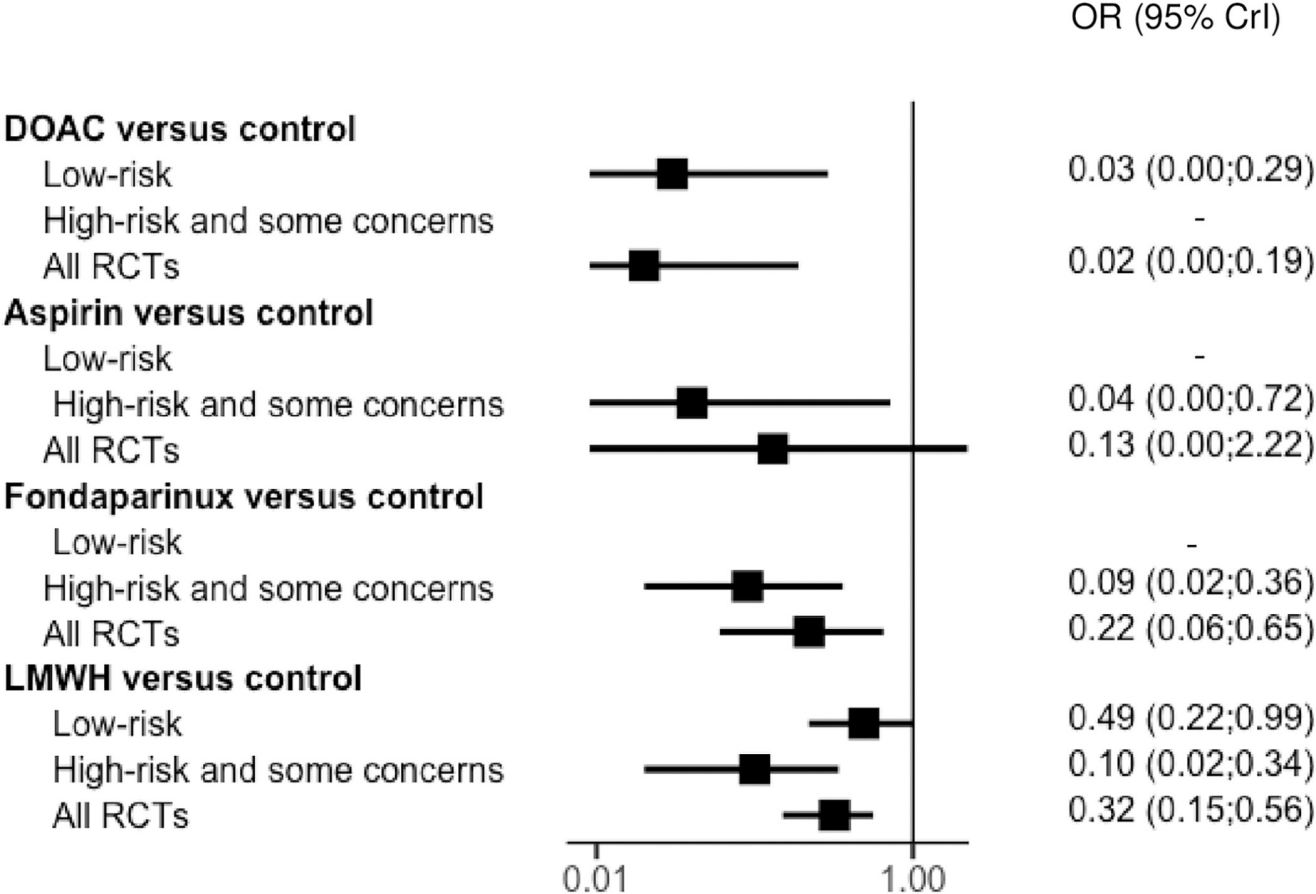
Network forest plot according to the risk of bias for the main outcome: major VTE with OR (points) and their 95% CrIs (lines). DOAC, direct oral anticoagulant; LMWH, low molecular weight heparin; OR, odds ratio; RCT, randomized controlled trial; VTE, venous thromboembolism; 95% CrI, 95% credible interval.

Thirteen trials reported outcomes for major VTE. Van Adrichem and colleagues did not perform a systematic assessment; as asymptomatic proximal DVT was not available, only symptomatic VTE were included for this study [3]. Without pharmacological thromboprophylaxis (control group), the rate of major VTE ranged from 0% to 11.7%, symptomatic VTE from 0% to 2.1%, and PE from 0% to 2.1%. Compared to the control group, rivaroxaban were associated with a significant risk reduction of major VTE in adults with lower limb immobilization after trauma (OR, 0.02; 95% CrI: 0.00 to 0.19), as well as fondaparinux (OR, 0.22; 95% CrI: 0.06 to 0.65) or LMWH (OR, 0.32, 95% CrI: 0.15 to 0.56) (Fig 3). No significant risk reduction of major VTE was shown with aspirin (OR, 0.13, 95% CrI: 0.00 to 2.22). When considering only low risk of bias studies, results are unchanged (Figs 4 and S3). No deaths were recorded in any of the studies except for the POT-CAST study, in which 1 death was reported to be related to PE.

**Fig 5:**
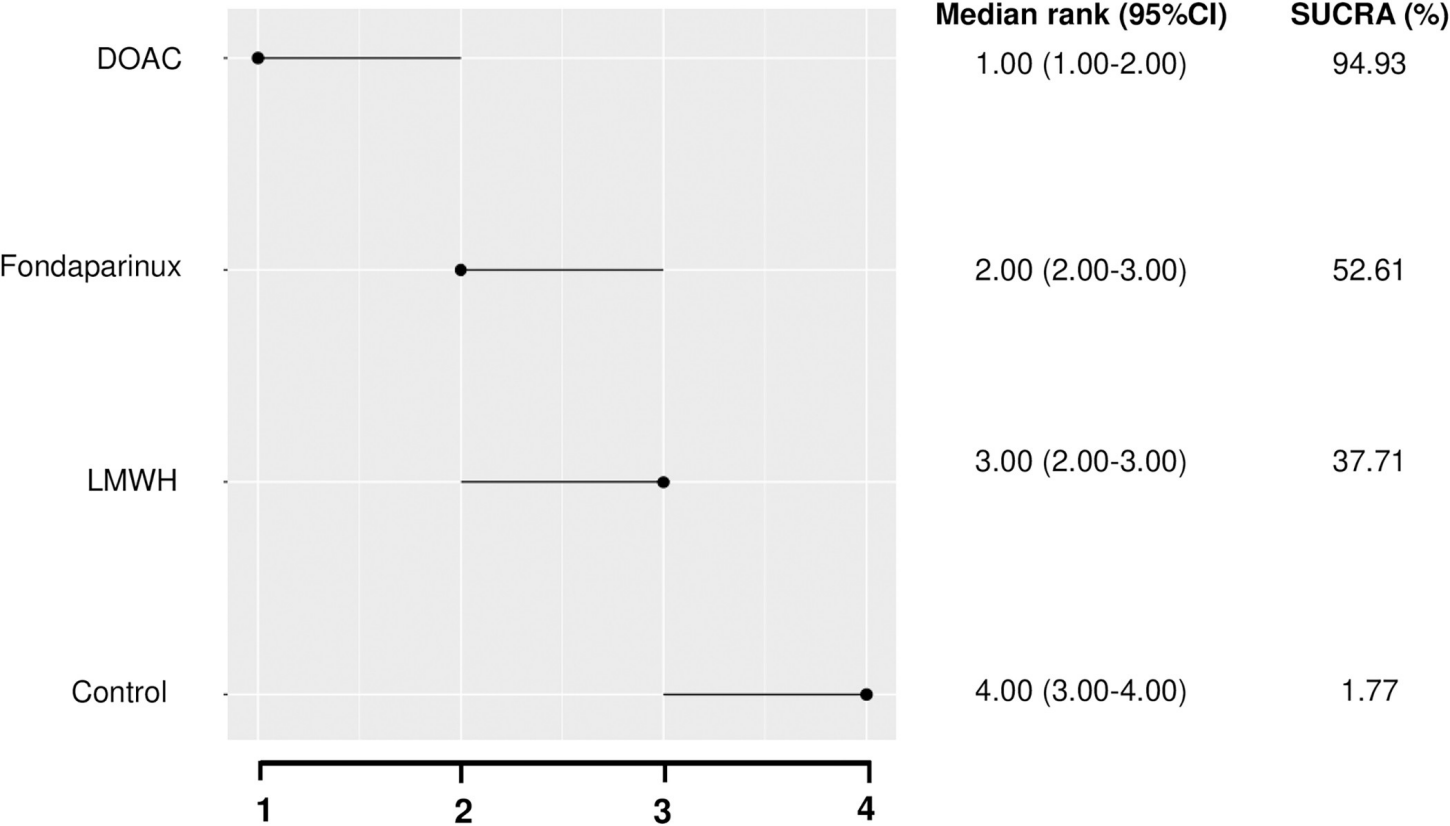
Median rank and SUCRA values of competing prophylactic treatments. DOAC, direct oral anticoagulant; LMWH, low molecular weight heparin; SUCRA, surface under the cumulative ranking curve.

The inconsistency of the model was tested on the direct comparison loop of LMWH, fondaparinux, and the control with a nonsignificant *p*-value (*p* = 0.335) allowing whole network estimates. Rivaroxaban is likely to be more effective than LMWH (OR, 0.07; 95% CrI: 0.00 to 0.56). This NMA did not detect a significant difference between the other treatments. Regarding the median rank, rivaroxaban is ranked first with a confidence interval between 1 to 2 (Fig 5). Presented in order, fondaparinux is ranked second (95% CrI 2 to 4) and LMWH is ranked third (95% CrI 2 to 4). When treatments were ranked, rivaroxaban had the highest likelihood of being ranked top in terms of efficacy (SUCRA = 94.9%), followed by fondaparinux (SUCRA = 52.6%) and LMWH (SUCRA = 37.7%). Aspirin was not considered effective and was not included in this ranking. The results of the network meta-regressions highlighted that none of the covariates (age, mean duration of treatment, rate of patients surgically treated, and study design: double blind versus others) improved the fit of the model in these analyses, which suggests that they did not modify the treatment effects.

In the sensitivity analysis with the symptomatic VTE endpoint, the magnitude effect was similar for the major VTE endpoint (i.e., for rivaroxaban (OR, 0.05; 95% CrI: 0.00 to 0.49), fondaparinux (OR, 0.19; 95% CrI: 0.03 to 0.97), and LMWH (OR, 0.41, 95% CrI: 0.12 to 0.98) (S4 Fig).

### Safety endpoint (major bleeding)

Data on major bleeding were available for 13 studies (*n* = 7,892). A total of 6 events occurred: none in the rivaroxaban, aspirin, and control groups, 5 among 3,556 patients in the LMWH group, and 1 among 3,466 patients in the fondaparinux group. A total of 9 patients had a nonmajor clinically relevant bleeding: 3 among 1,078 patients in the rivaroxaban group, 5 among 3,353 patients among the LMWH group, and 1 among 766 patients in the fondaparinux group. Given the very low risk of major bleeding, no model was built to evaluate this endpoint and no comparison of treatment by ranking was performed.

### Net clinical benefit (major VTE and major bleeding)

**Fig 6:**
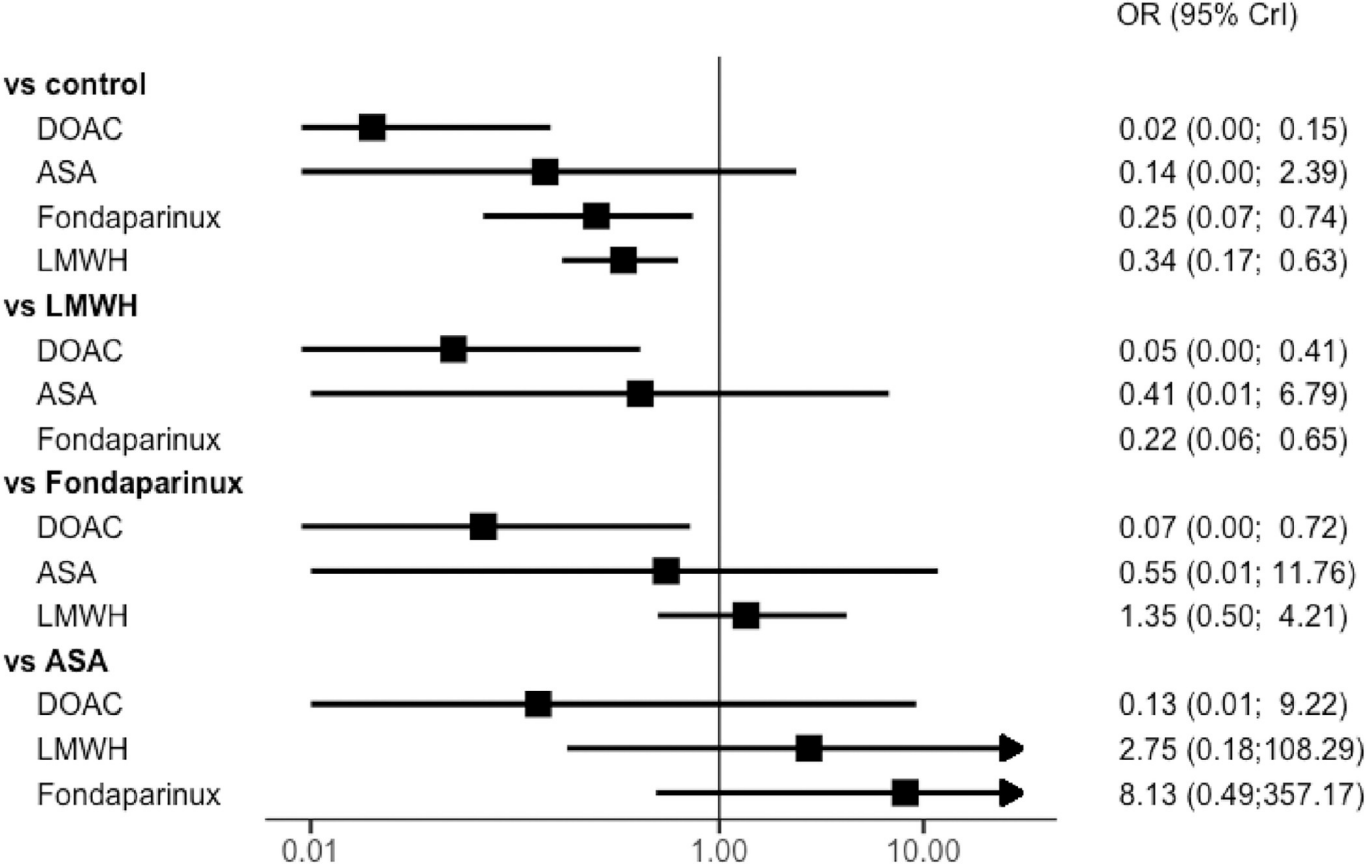
Network forest plot for the secondary outcome (net clinical benefit: major VTE and major bleeding) in all RCTs with OR (points) and their 95% CrIs (lines). ASA, aspirin; DOAC, direct oral anticoagulant; LMWH, low molecular weight heparin; OR, odds ratio; RCT, randomized controlled trial; VTE, venous thromboembolism; 95% CrI, 95% credible interval.

Compared to the control group, rivaroxaban was associated with an increase of net clinical benefit in adult with lower limb immobilization after trauma (OR, 0.02; 95% CrI: 0.00 to 0.15), as well as fondaparinux (OR, 0.25; 95% CrI: 0.07 to 0.74) and LMWH (OR, 0.34, 95% CrI: 0.17 to 0.63) (Fig 6). Rivaroxaban was likely to be more effective than LMWH (OR, 0.05, 95% CrI: 0.00 to 0.41) and fondaparinux (OR, 0.07, 95% CrI: 0.00 to 0.72).

## Discussion

This NMA including 14 studies evaluating the efficacy and the safety of 4 anticoagulant treatments in the prevention of venous thromboembolic events in more than 8,000 patients immobilized after trauma shows that rivaroxaban, fondaparinux, and LMWHs reduce the risk of major VTE compared with a placebo or no treatment. Considering only symptomatic VTE, the results remain unchanged but with wider confidence intervals due to reduced power. These results were unchanged when including only low risk of bias studies, even if the magnitude of the effect of LMWH is less convincing. These results about LMWH are consistent with the other meta-analyses [38,39].

Of the DOACs, only rivaroxaban has been evaluated. This treatment was ranked first in terms of efficacy with no statistical excess risk of bleeding. A Bayesian NMA evaluating different thromboprophylaxis during hip or knee replacement surgery showed an identical ranking of molecules with rivaroxaban in the lead [40].

Prophylaxis in patients with lower extremity trauma is a particular and rather unique situation. The net clinical benefit endpoint is dominated by the risk of major VTE, the rate of major bleeding being much lower than the risk of VTE. That is not very surprising since the median age is around 48 years, whereas in other types of primary prevention of VTE, patients are and therefore more at risk of bleeding but also because their clinical conditions are not associated with bleeding risk such as major surgical procedures, hospitalization for acute disease, or intensive care units.

The benefit–risk profile of these treatments is to date not questionable, except in terms of cost-effectiveness and patient preference. And beyond the question of the benefit that seems to be in favor, it is now necessary to define which one to choose.

The place of aspirin for thromboprophylaxis of patients with immobilization after injury remains a matter of debate. It is therefore not possible to answer its place in the therapeutic arsenal. Aspirin is indicated for primary prophylaxis in patients undergoing major orthopedic surgery (i.e., total hip or knee replacement) [15,41]. Aspirin must now be evaluated in this indication of patients with immobilization in the context of trauma. Regarding rivaroxaban, PRONOMOS was the first study to assess their efficacy and safety in nonmajor surgery patients. Apart from 2 observational studies with methodological weaknesses, there is no study evaluating DOAC in this large population of lower extremity trauma patients requiring immobilization without surgery [7,42]. In sum, although the ranking allows to compare the effect of the 3 different agents that were significantly effective versus control (rivaroxaban, LMWH, and fondaparinux), this ranking remains imprecise. High-level evidence studies, mainly for oral treatments, are now necessary to guide future guidelines in this population [43,44].

This NMA is an update of the first one performed by Horner and colleagues. The creation of a living meta-analysis on this topic would be very useful to carry on these analyses. Living network meta-analyses allow for a broad, comprehensive and up-to-date presentation of evidence [45,46]. This living meta-analysis could lead to living clinical guidelines [46].

The main strength of this study is that it has synthesized data from more than 8,000 participants in 14 RCTs. This represents a large, methodologically robust method to simultaneously estimate of relative treatment effects. Furthermore, the analysis was performed with a Bayesian approach allowing the comparison and ranking of treatments between them. The analysis of individual data from the PRONOMOS study provided the first opportunity to assess DOAC in this indication through a high-level evidence RCT. The meta-analysis provides a global answer, but in the era of personalized treatment, patient preference and physician experience remain important in deciding whether or not to use prophylaxis based on baseline risk, estimated by medical experience or a risk score [1,44,47], and depending on the type of surgery and patient characteristics.

Some limitations need to be discussed. First, among the 14 studies performed in patients with lower extremity immobilization following trauma and eligible for this NMA, only 50% of the studies are considered at low risk of bias (i.e., with adequate allocation concealment and blinded outcome assessment). However, we reported small differences in terms of treatment effects estimated from RCTs at low risk of bias and all RCTs. Another limitation is that the network had as many indirect comparisons as direct comparisons. For example, aspirin is only linked by an indirect comparison to DOAC, fondaparinux, and a control. To date, there are few data on the efficacy of aspirin in this indication (144 patients). Furthermore, the definitions of major VTE were not consensual and the choice of this definition may have underestimated the event rate in both arms of the studies that considered only symptomatic events (i.e., did not assess asymptomatic proximal DVT). Finally, among DOAC, only rivaroxaban was assessed, and our findings may be extrapolated to others, albeit with caution.

## Conclusions

This NMA shows the efficacy of rivaroxaban, fondaparinux, and LMWH in preventing VTE in patients with lower extremity trauma requiring immobilization, compared with placebo or no treatment. Rivaroxaban has the highest likelihood of being top ranked in terms of efficacy and net clinical benefit. However, the decision to initiate thromboprophylaxis must also consider cost-effectiveness, cost-utility, baseline risk of each patient, estimated by medical experience or a risk score and patient preference.

## Supporting information

S1 PRISMA Checklist Preferred Reporting Items for Systematic reviews and Meta-Analyses extension for Scoping Reviews (PRISMA-ScR) Checklist. (DOCX)

S1 Fig Risk of bias assessment summary. (PDF)

S2 Fig Risk of bias assessment graph. (PDF)

S3 Fig Median rank and SUCRA values of competing prophylactic treatments for only RCT with low risk of bias for the primary outcome (major VTE). RCT, randomized controlled trial; SUCRA, surface under the cumulative ranking curve; VTE, venous thromboembolism.
(PDF)

S4 Fig Network Forest plot for the secondary outcome (symptomatic VTE) in all RCTs with OR (points) and their 95% CrIs (lines). ASA, aspirin; DOAC, direct oral anticoagulant; LMWH, low molecular weight heparin; OR, odds ratio; RCT, randomized controlled trial; VTE, venous thromboembolism; 95% CrI, 95% credible interval.
(PDF)

S1 Table Literature search strategies. (DOCX)

S2 Table Excluded studies with rationale. (DOCX)

## Abbreviations

DOAC: direct oral anticoagulant
DVT: deep vein thrombosis
ISTH: International Society on Thrombosis and Haemostasis
LMWH: low molecular weight heparin
NMA: network meta-analysis
OR: odds ratio; PE, pulmonary embolism
RCT: randomized controlled trial
ROBINS-I: risk of bias in nonrandomized studies—of interventions
SUCRA: surface under the cumulative ranking curve
VTE: venous thromboembolism; 95% CrI, 95% credible interval
