# Supplementary material for: Prevention of venous thromboembolic events in patients with lower leg immobilization after trauma: Systematic review and network meta-analysis with meta-epsidemiological approach
Source: PLoS Med. 2022 Jul 18;19(7):e1004059. doi: 10.1371/journal.pmed.1004059 (PMC9342742; doi:10.1371/journal.pmed.1004059)
Supplement: S1 Fig — (PDF) [file pmed.1004059.s002.pdf]

	Bias arising from the randomization process	Bias due to deviations from the intended interventions	Missing outcome data	Bias in measurement of the outcome	Bias in selection of the reported result	Overall risk of bias✷
Kujath et al, 1993	C	C	+	+	C	+
Kock et al, 1995	C	C	+	+	C	+
Gehling et al, 1998	C	C	–	+	C	+
Lassen et al, 2002	–	–	–	–	C	C
Jørgensen et al, 2002	–	C	–	–	C	C
Lapidus et al, 2007a	–	–	–	–	C	C
Lapidus et al, 2007b	–	–	–	–	C	C
Goel et al, 2009	–	–	–	–	C	C
Samama et al, 2013	–	C	–	–	C	C
Selby et al, 2015	–	–	–	–	C	C
Zheng et al, 2016	C	+	+	–	C	+
Bruntink et al, 2017	–	+	–	–	C	+
Van Adrichem et al, 2017	–	–	–	–	C	C
Samama et al, 2020	–	–	–	–	–	–

– Low risk of Biais

C Some Concerns

+ High risk of Biais

✷ Overall risk-of-bias judgement: For low risk of bias, the study is judged to be at low risk of bias for all domains for this result; for some concerns, The study is judged to raise some concerns in at least one domain for this result, but not to be at high risk of bias for any domain, for high risk of bias, the study is judged to be at high risk of bias in at least one domain for this result or the study is judged to have some concerns for multiple domains in a way that substantially lowers confidence in the result.

a: Ankle fracture

b: Achille tendon rupture
