# Supplementary figures and images for: Prevention of venous thromboembolic events in patients with lower leg immobilization after trauma: Systematic review and network meta-analysis with meta-epsidemiological approach
Source: PLoS Med. 2022 Jul 18;19(7):e1004059. doi: 10.1371/journal.pmed.1004059 (PMC9342742; doi:10.1371/journal.pmed.1004059)

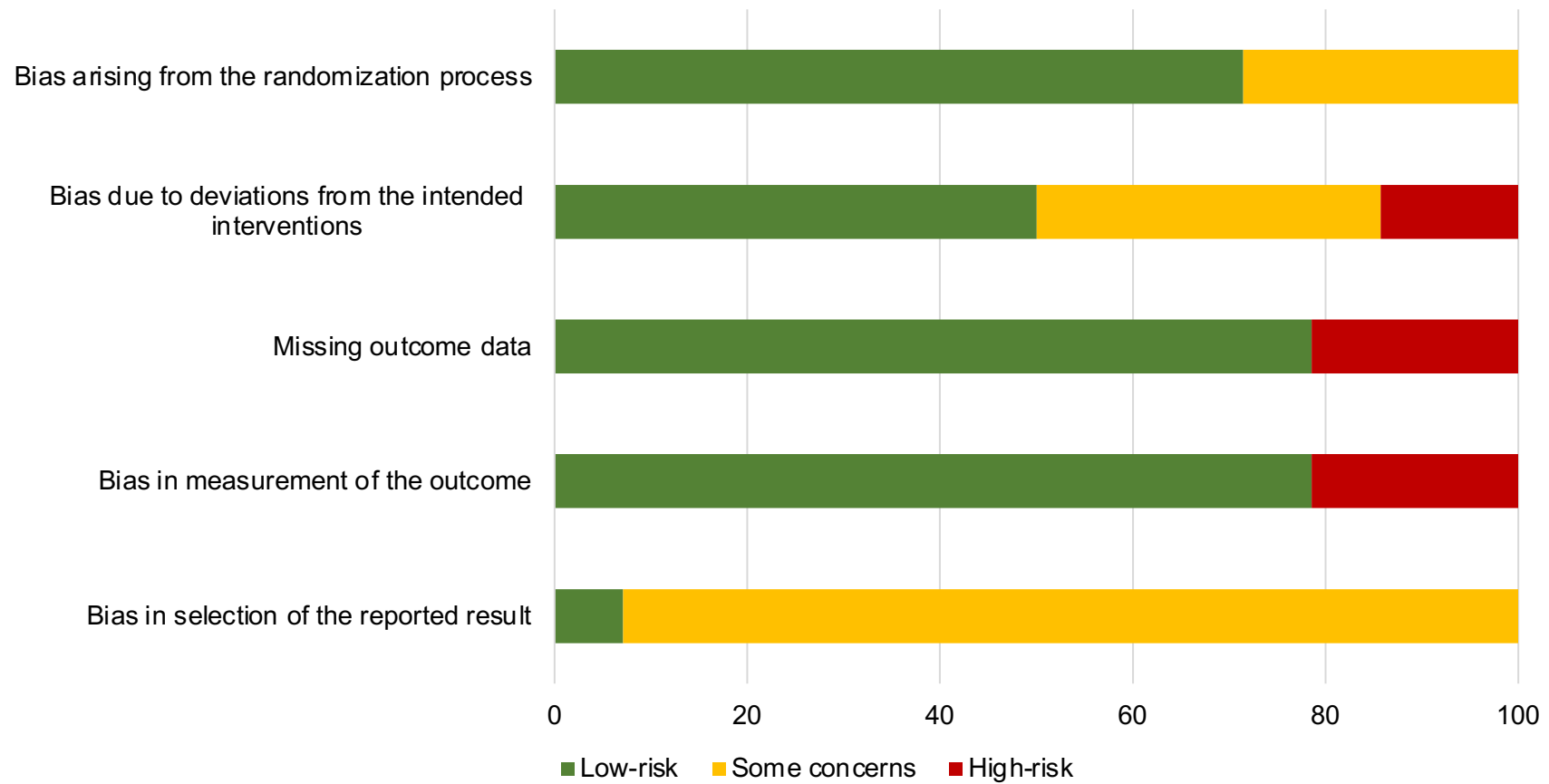

Figure E2. Risk of bias assessment graph.

Supplement: S2 Fig — (PDF) [file pmed.1004059.s003.pdf]
