# Supplementary material for: Prevention of venous thromboembolic events in patients with lower leg immobilization after trauma: Systematic review and network meta-analysis with meta-epsidemiological approach
Source: PLoS Med. 2022 Jul 18;19(7):e1004059. doi: 10.1371/journal.pmed.1004059 (PMC9342742; doi:10.1371/journal.pmed.1004059)
Supplement: S3 Fig — RCT, randomized controlled trial; SUCRA, surface under the cumulative ranking curve; VTE, venous thromboembolism. (PDF) [file pmed.1004059.s004.pdf]

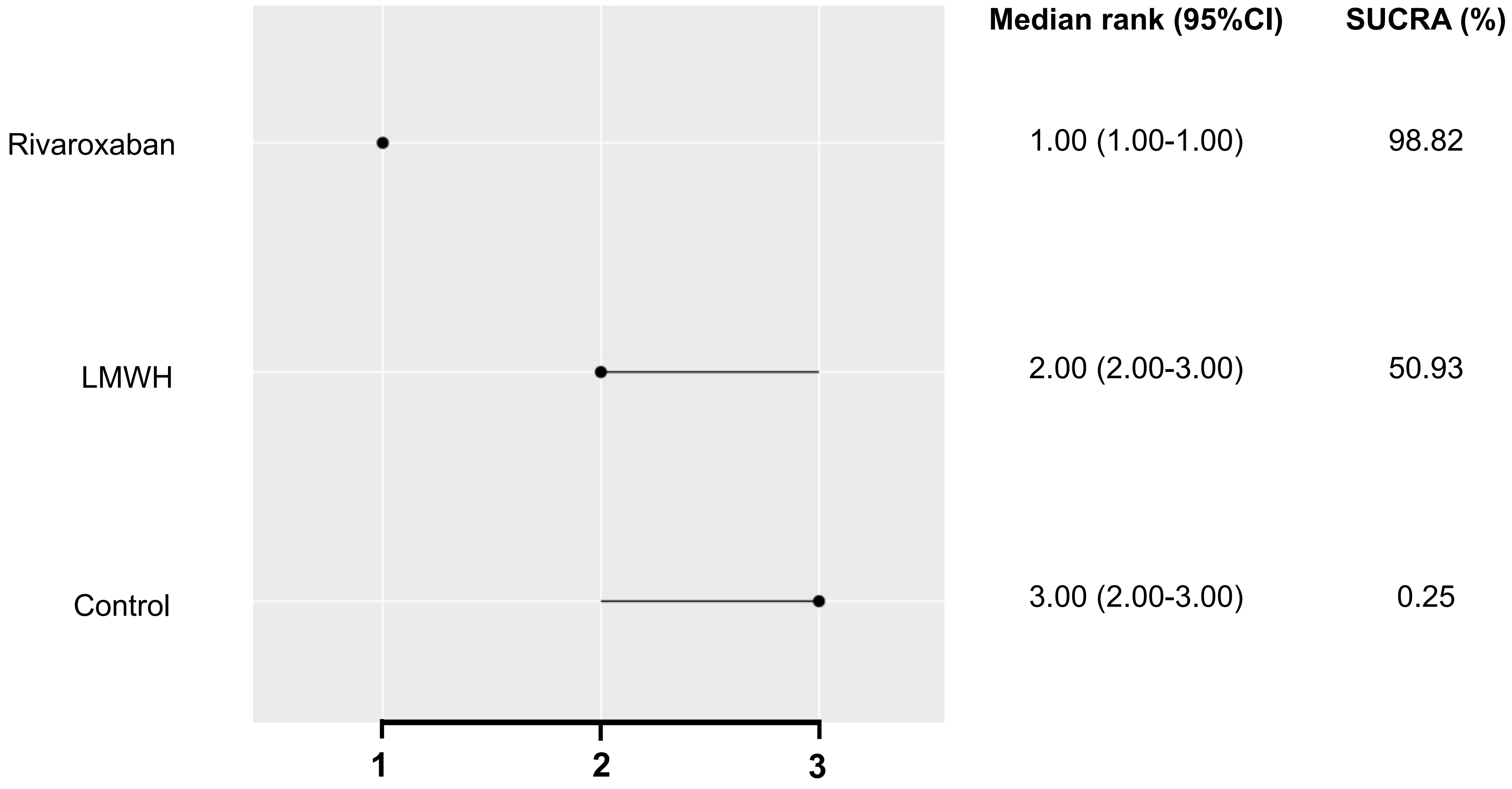

Figure E3. Median rank and SUCRA (surface under the cumulative ranking) values of competing prophylactic treatments for only RCT with low risk of bias for the primary outcome (major VTE).
