# Supplementary material for: Prevention of venous thromboembolic events in patients with lower leg immobilization after trauma: Systematic review and network meta-analysis with meta-epsidemiological approach
Source: PLoS Med. 2022 Jul 18;19(7):e1004059. doi: 10.1371/journal.pmed.1004059 (PMC9342742; doi:10.1371/journal.pmed.1004059)
Supplement: S4 Fig — ASA, aspirin; DOAC, direct oral anticoagulant; LMWH, low molecular weight heparin; OR, odds ratio; RCT, randomized controlled trial; VTE, venous thromboembolism; 95% CrI, 95% credible interval. (PDF) [file pmed.1004059.s005.pdf]

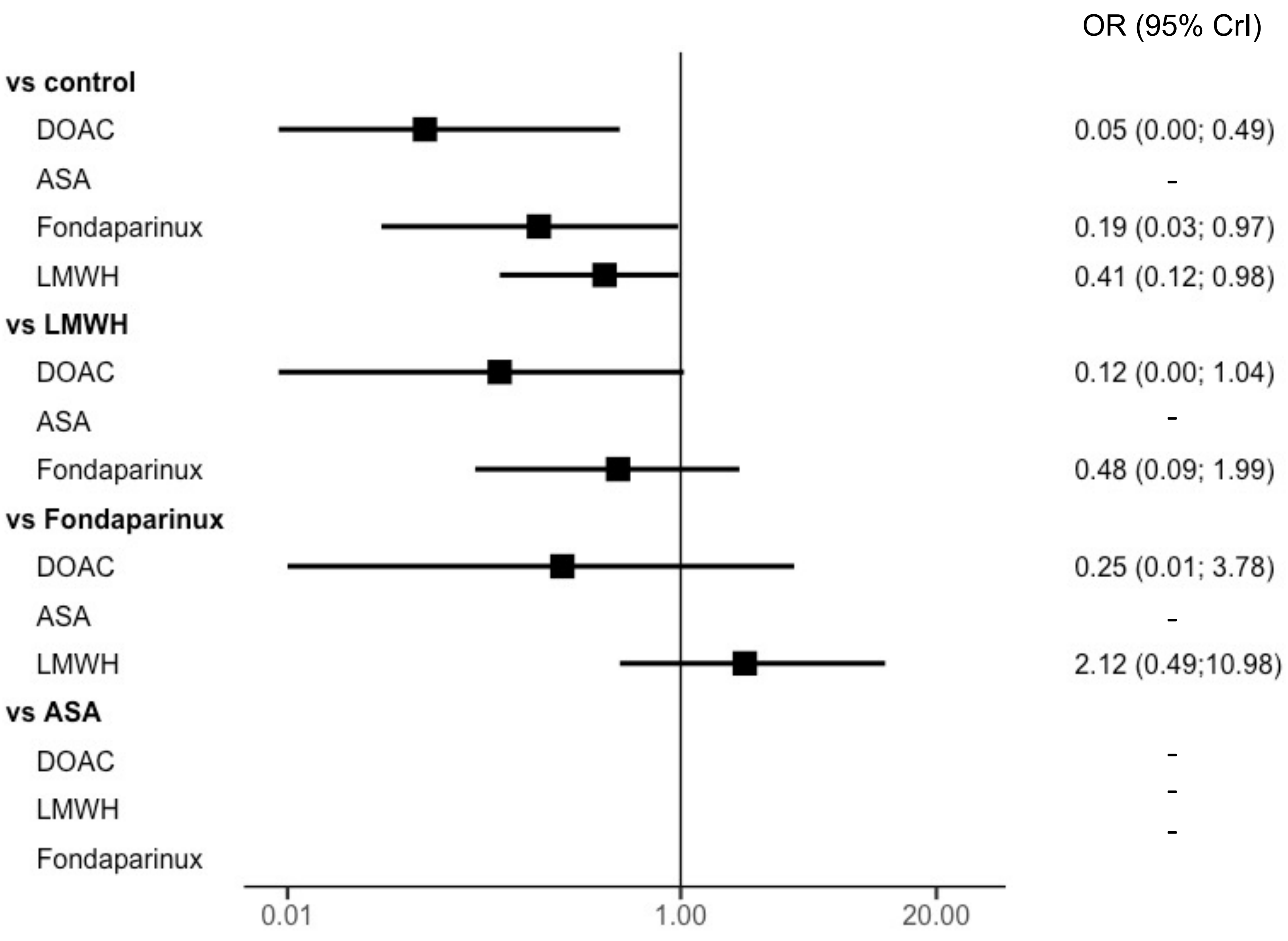

Figure E4. Network forest plot for the secondary outcome (symptomatic venous thromboembolism) in all randomized controlled trials with odds ratio (points) and their 95%CrIs (lines).
