# Supplementary material for: Prevention of venous thromboembolic events in patients with lower leg immobilization after trauma: Systematic review and network meta-analysis with meta-epsidemiological approach
Source: PLoS Med. 2022 Jul 18;19(7):e1004059. doi: 10.1371/journal.pmed.1004059 (PMC9342742; doi:10.1371/journal.pmed.1004059)
Supplement: S1 Table — (DOCX) [file pmed.1004059.s006.docx]

**Table E1. Literature search strategies.**

**Database searched: National Library of Medicine, pubmed, MEDLINE**

**Date of coverage:** January 1990 to May 2021

**Search undertaken:** May 2021

#1 (("venous thrombosis"[MeSH Terms] OR "venous thrombosis"[MeSH Terms] OR "venous thromboembolism"[MeSH Terms] OR "venous thrombosis"[MeSH Terms] OR "venous thrombosis"[MeSH Terms] OR "pulmonary embolism"[MeSH Terms] OR "thromboprophyla*"[Title/Abstract] OR "thrombus*"[Title/Abstract] OR "thrombotic*"[Title/Abstract] OR "thrombolic*"[Title/Abstract] OR "thromboemboli*"[Title/Abstract])

#2 (("splint"[Title/Abstract] OR "cast*"[Title/Abstract] OR "cast"[Title/Abstract] OR "immobili*"[Title/Abstract] OR "immobilisation"[Title/Abstract] OR "immobilization"[Title/Abstract] OR "brace"[Title/Abstract] OR "braces"[Title/Abstract] OR "mobility limitation"[Title/Abstract] OR "trauma"[Title/Abstract] OR "injury"[Title/Abstract] OR "fracture"[Title/Abstract] OR "ankle"[Title/Abstract] OR "lower limb"[Title/Abstract] OR "leg"[Title/Abstract])

#3 (("anticoagulants"[MeSH Terms] OR "aspirin"[MeSH Terms] OR "aspirin"[MeSH Terms] OR "heparin, low molecular weight"[MeSH Terms] OR "heparin, low molecular weight"[MeSH Terms] OR "DOAC"[Title/Abstract] OR "NOAC"[Title/Abstract] OR "direct oral anticoagulant"[Title/Abstract] OR "rivaroxaban"[Title/Abstract] OR "pradaxa"[Title/Abstract] OR "apixaban"[Title/Abstract] OR "eliquis"[Title/Abstract] OR "acetylsalicylic acid"[Title/Abstract] OR "aspirin"[Title/Abstract] OR "edoxaban"[Title/Abstract] OR "clivarin"[Title/Abstract] OR "reviparin"[Title/Abstract] OR "certoparin"[Title/Abstract] OR "certoparin"[Title/Abstract] OR "innohep"[Title/Abstract] OR "tinzaparin"[Title/Abstract] OR "fragmin"[Title/Abstract] OR "dalteparin"[Title/Abstract] OR "nadroparin"[Title/Abstract] OR "enoxaparin"[Title/Abstract] OR "fraxiparin"[Title/Abstract])

#4 ("classical article"[Publication Type] OR "clinical study"[Publication Type] OR "clinical trial"[Publication Type] OR "clinical trial, phase iv"[Publication Type] OR "comparative study"[Publication Type] OR "controlled clinical trial"[Publication Type] OR "english abstract"[Publication Type] OR "evaluation study"[Publication Type] OR "journal article"[Publication Type] OR "letter"[Publication Type] OR "multicenter study"[Publication Type] OR "observational study"[Publication Type] OR "preprint"[Publication Type] OR "randomized controlled trial"[Publication Type] OR "validation study"[Publication Type])

#5: "arthroplasty, replacement, hip"[MeSH Terms]) NOT ("case reports"[Publication Type])

#6: #1 AND #2 AND #3 AND #4 NOT #5

n=2,270
