# Supplementary material for: Prevention of venous thromboembolic events in patients with lower leg immobilization after trauma: Systematic review and network meta-analysis with meta-epsidemiological approach
Source: PLoS Med. 2022 Jul 18;19(7):e1004059. doi: 10.1371/journal.pmed.1004059 (PMC9342742; doi:10.1371/journal.pmed.1004059)
Supplement: S2 Table — (DOCX) [file pmed.1004059.s007.docx]

**S2 Table. Excluded studies with rationale**

|  | **Author, year** | **Year** | **Reasons for exclusion** |
| --- | --- | --- | --- |
|  | Ahmad J *et al*.^1^ | 2017 | No information on treatment received. |
|  | Ajwani SH *et al*.^2^ | 2016 | No information on treatment received. |
|  | Ali IA *et al*. ^3^ | 2021 | No consideration of confounding factors. |
|  | Bandle J *et al*.^4^ | 2014 | Survey report. |
|  | Baraza N *et al*.^5^ | 2013 | No information on VTE events. |
|  | Bertoletti L *et al*.^6^ | 2011 | No information on treatment received. |
|  | Blanco JA *et al*.^7^ | 2018 | Allocation of treatment according to patient characteristics |
|  | Braithwaite I *et al*.^8^ | 2016 | No consideration of confounding factors. |
|  | Brill JB *et al*.^9^ | 2016 | Single case-control study. After discussion DD, CC, SL, choice to exclude. |
|  | Choufani C *et al*.^10^ | 2018 | Inclusion of patients for air travel and no accurate information on treatments received. |
|  | Çolak İ *et al*.^11^ | 2020 | No information on treatment received. |
|  | Davis S *et al*.^12^ | 2019 | No information on treatment received. |
|  | Eisele R *et al*. ^13^ | 2001 | Allocation of treatment according to patient characteristics |
|  | Engler ID *et al*.^14^ | 2019 | No clear information on the treatments received. |
|  | Griffiths JT *et al*.^15^ | 2012 | Population: only elective surgery, no trauma context. |
|  | Haac BE *et al*.^16^ | 2020 | Population: major trauma/polytrauma. |
|  | Haac BE *et al*.^17^ | 2017 | Population: inclusion of upper limb trauma (25%). |
|  | Haque S *et al*.^18^ | 2015 | Allocation of treatment according to patient characteristics |
|  | Haque S *et al*.^19^ | 2016 | No information on treatment received. |
|  | Heijboer RRO *et al*.^20^ | 2019 | No information on the context of trauma or not. |
|  | Heijboer RRO *et al*.^21^ | 2019 | No clear information on the treatments received. |
|  | Heyes GJ *et al*.^22^ | 2015 | No treatment received. |
|  | Hunter AM *et al*.^23^ | 2020 | No consideration of confounding factors. |
|  | Jameson SS *et al*.^24^ | 2011 | No information on treatment received. |
|  | Kopelman TR *et al*.^25^ | 2013 | Population: major trauma/polytrauma. |
|  | Little MT *et al*.^26^ | 2016 | Commentary report. |
|  | Menakaya CU *et al*.^27^ | 2014 | No clear information on the treatments received. |
|  | Menakaya CU *et al*.^28^ | 2013 | Medico-economic study and no clear information on the treatments received. |
|  | Peláez-Damy P *et al*.^29^ | 2017 | No clear information on VTE events. |
|  | Riou B *et al*.^30^ | 2007 | Allocation of treatment according to patient characteristics (gestalt) |
|  | Shibuya N *et al*.^31^ | 2012 | No information on treatment received. |
|  | Starr AJ *et al*.^32^ | 2019 | Population: major trauma/polytrauma. |
|  | Walenga JM *et al*.^33^ | 2014 | Substudy of an included full text study (Lassen et al.,2002^34^) |
|  | Wang PF *et al*.^35^ | 2021 | Population: More than 50% are patients with pelvic and femoral neck fractures |
|  | Zhang BF *et al*.^36^ | 2020 | Population: 65% of patients had a hip fracture |
|  | Zhang W *et al*.^37^ | 2019 | No information on treatment received. |

**References:**

1. Ahmad J, Lynch M-K, Maltenfort M. Incidence and Risk Factors of Venous Thromboembolism After Orthopaedic Foot and Ankle Surgery. :6.

2. Ajwani SH, Shaw A, Naiz O, Bhaskar D, Charalambous CP. Venous Thromboembolism Prophylaxis in Outpatient Lower Limb Fractures and Injuries. :6.

3. Ali IA, Elbana H, Farhan M, Qureshi IF, Mubashir A. Venous thromboembolism in trauma patients with lower limb cast immobilization, associated risk reduction and complication using rivaroxaban. *Ir J Med Sci*. 2021;190(1):169-175. doi:10.1007/s11845-020-02306-3

4. Bandle J, Shackford SR, Sise CB, Knudson MM. Variability is the standard: The management of venous thromboembolic disease following trauma. *Journal of Trauma and Acute Care Surgery*. 2014;76(1):213-216. doi:10.1097/TA.0b013e3182aa2fa9

5. Baraza N, Lever S, Dhukaram V. Home therapy pathway – Safe and streamlined method of initial management of ankle fractures. *Foot and Ankle Surgery*. 2013;19(4):250-254. doi:10.1016/j.fas.2013.06.014

6. Bertoletti L, Righini M, Bounameaux H, et al. Acute venous thromboembolism after non-major orthopaedic surgery or post-traumatic limb immobilisation: Findings from the RIETE registry. *Thromb Haemost*. 2011;105(04):739-741. doi:10.1160/TH10-11-0751

7. Blanco JA, Slater G, Mangwani J. A Prospective Cohort Study of Symptomatic Venous Thromboembolic Events in Foot and Ankle Trauma: The Need for Stratification in Thromboprophylaxis? *The Journal of Foot and Ankle Surgery*. 2018;57(3):484-488. doi:10.1053/j.jfas.2017.10.036

8. Braithwaite I, Dunbar L, Eathorne A, Weatherall M, Beasley R. Venous thromboembolism rates in patients with lower limb immobilization after Achilles tendon injury are unchanged after the introduction of prophylactic aspirin: audit. *J Thromb Haemost*. 2016;14(2):331-335. doi:10.1111/jth.13224

9. Brill JB, Calvo RY, Wallace JD, et al. Aspirin as added prophylaxis for deep vein thrombosis in trauma: A retrospective case-control study. *Journal of Trauma and Acute Care Surgery*. 2016;80(4):625-630. doi:10.1097/TA.0000000000000977

10. Choufani C. Risque thromboembolique chez les patients traumatisés des membres inférieurs rapatriés. :5.

11. Çolak İ, Gülabi D, Eceviz E, Çevik BH, Bulut G, Bekler HI. Incidence of Venous Thromboembolism After Achilles Tendon Surgery in Patients Receiving Thromboprophlaxis. *Journal of the American Podiatric Medical Association*. 2020;110(Article_3). doi:10.7547/17-012

12. Davis S, Goodacre S, Pandor A, et al. Decision‐analysis modelling of the effects of thromboprophylaxis for people with lower limb immobilisation for injury. *Br J Haematol*. 2019;186(1):166-168. doi:10.1111/bjh.15748

13. Eisele R, Greger W, Weikert E, Kinzl L. [Ambulatory prevention of thrombosis in traumatology]. *Unfallchirurg*. 2001;104(3):240-245. doi:10.1007/s001130050720

14. Engler ID, Bragg JT, Miller SL. Incidence of Deep Venous Thrombosis Associated With Proximal Hamstring Rupture. :4.

15. Griffiths JT, Matthews L, Pearce CJ, Calder JDF. Incidence of venous thromboembolism in elective foot and ankle surgery with and without aspirin prophylaxis. *The Journal of Bone and Joint Surgery British volume*. 2012;94-B(2):210-214. doi:10.1302/0301-620X.94B2.27579

16. Haac BE, O’Hara NN, Mullins CD, et al. Patient preferences for venous thromboembolism prophylaxis after injury: a discrete choice experiment. *BMJ Open*. 2017;7(8):e016676. doi:10.1136/bmjopen-2017-016676

17. Haac BE, O’Hara NN, Manson TT, et al. Aspirin versus low-molecular-weight heparin for venous thromboembolism prophylaxis in orthopaedic trauma patients: A patient-centered randomized controlled trial. Leroyer C, ed. *PLoS ONE*. 2020;15(8):e0235628. doi:10.1371/journal.pone.0235628

18. Haque S, Davies MB. Oral thromboprophylaxis in patients with ankle fractures immobilized in a below the knee cast. *Foot and Ankle Surgery*. 2015;21(4):266-268. doi:10.1016/j.fas.2015.02.002

19. Haque S, Bishnoi A, Khairandish H, Menon D. Thromboprophylaxis in Ambulatory Trauma Patients With Foot and Ankle Fractures: Prospective Study Using a Risk Scoring System. *Foot & Ankle Specialist*. 2016;9(5):388-393. doi:10.1177/1938640016640892

20. Heijboer RRO, Lubberts B, Guss D, Johnson AH, Moon DK, DiGiovanni CW. Venous Thromboembolism and Bleeding Adverse Events in Lower Leg, Ankle, and Foot Orthopaedic Surgery with and without Anticoagulants. *The Journal of Bone and Joint Surgery*. 2019;101(6):539-546. doi:10.2106/JBJS.18.00346

21. Heijboer RRO, Lubberts B, Guss D, Johnson AH, DiGiovanni CW. Incidence and Risk Factors Associated with Venous Thromboembolism After Orthopaedic Below-knee Surgery: *Journal of the American Academy of Orthopaedic Surgeons*. 2019;27(10):e482-e490. doi:10.5435/JAAOS-D-17-00787

22. Heyes GJ, Tucker A, Michael ALR, Wallace RGH. The incidence of deep vein thrombosis and pulmonary embolism following cast immobilisation and early functional bracing of Tendo Achilles rupture without thromboprophylaxis. :4.

23. Hunter AM, Montgomery TP, Pitts CC, et al. Postoperative aspirin use and its effect on bone healing in the treatment of ankle fractures. *Injury*. 2020;51(2):554-558. doi:10.1016/j.injury.2019.11.039

24. Jameson SS, Augustine A, James P, et al. Venous thromboembolic events following foot and ankle surgery in the English National Health Service. *THE JOURNAL OF BONE AND JOINT SURGERY*. 2011;93(4):8.

25. Kopelman TR, O’Neill PJ, Pieri PG, et al. Alternative dosing of prophylactic enoxaparin in the trauma patient: is more the answer? *Am J Surg*. 2013;206(6):911-915; discussion 915-916. doi:10.1016/j.amjsurg.2013.10.005

26. Little MTM. Low-Molecular-Weight Heparin Did Not Differ from Placebo in Preventing Clinically Important Deep Venous Thrombosis After Surgical Repair of Leg Fracture. *The Journal of Bone and Joint Surgery*. 2016;98(4):316. doi:10.2106/JBJS.15.01442

27. Menakaya CU, Boddice T, Malhotra R, et al. Outpatients thromboprophylaxis following lower limb immobilisation: an institution’s experience. *Eur Orthop Traumatol*. 2014;5(4):335-339. doi:10.1007/s12570-013-0240-0

28. Menakaya CU, Pennington N, Muthukumar N, et al. The cost of outpatient venous thromboembolism prophylaxis following lower limb injuries. *The Bone & Joint Journal*. 2013;95-B(5):673-677. doi:10.1302/0301-620X.95B5.30555

29. Peláez-Damy P, Torres-Gómez A, Mas-Celis F, Martínez-Villalobos M. A comparative study in patients with fractures of the lower limbs between rivaroxaban versus enoxaparin and its impact on bone healing time. *Acta Ortop Mex*. 2017;31(4):177-181.

30. Riou B, Rothmann C, Lecoules N, et al. Incidence and risk factors for venous thromboembolism in patients with nonsurgical isolated lower limb injuries. *The American Journal of Emergency Medicine*. 2007;25(5):502-508. doi:10.1016/j.ajem.2006.09.012

31. Shibuya N, Frost CH, Campbell JD, Davis ML, Jupiter DC. Incidence of Acute Deep Vein Thrombosis and Pulmonary Embolism in Foot and Ankle Trauma: Analysis of the National Trauma Data Bank. *The Journal of Foot and Ankle Surgery*. 2012;51(1):63-68. doi:10.1053/j.jfas.2011.10.017

32. Starr AJ, Shirley Z, Sutphin PD, et al. Significant Reduction of Pulmonary Embolism in Orthopaedic Trauma Patients. *J Orthop Trauma*. 2019;33(2):78-81. doi:10.1097/BOT.0000000000001346

33. Walenga JM, Kaiser PC, Prechel MM, et al. Sustained Release of Tissue Factor Following Thrombosis of Lower Limb Trauma. *Clin Appl Thromb Hemost*. 2014;20(7):678-686. doi:10.1177/1076029614545212

34. Lassen MR, Borris LC, Nakov RL. Use of the low-molecular-weight heparin reviparin to prevent deep-vein thrombosis after leg injury requiring immobilization. *N Engl J Med*. 2002;347(10):726-730. doi:10.1056/NEJMoa011327

35. Wang P-F, Zhang B-F, Xue H, et al. The Incidence and Location of Deep Vein Thrombosis in Lower Extremity Fracture Patients Receiving Sequential Chemical Prophylaxis. *Clin Appl Thromb Hemost*. 2021;27:107602962098763. doi:10.1177/1076029620987630

36. Zhang B-F, Wang P-F, Fei C, et al. Perioperative Deep Vein Thrombosis in Patients With Lower Extremity Fractures: An Observational Study. *Clin Appl Thromb Hemost*. 2020;26:107602962093027. doi:10.1177/1076029620930272

37. Zhang W, Huai Y, Wang W, et al. A Retrospective cohort study on the risk factors of deep vein thrombosis (DVT) for patients with traumatic fracture at Honghui Hospital. *BMJ Open*. 2019;9(3). doi:10.1136/bmjopen-2018-024247
